# Palladium(0) Catalyzed Synthesis of (*E*)-4-Bromo-*N*-((3-bromothiophen-2-yl)methylene)-2-methylaniline Derivatives via Suzuki Cross-Coupling Reaction: An Exploration of Their Non-Linear Optical Properties, Reactivity and Structural Features

**DOI:** 10.3390/molecules26185605

**Published:** 2021-09-15

**Authors:** Komal Rizwan, Nasir Rasool, Muhammad Ali Hashmi, Sadia Noreen, Muhammad Zubair, Mahwish Arshad, Syed Adnan Ali Shah

**Affiliations:** 1 Department of Chemistry, Government College University, Faisalabad 38000, Pakistan; Komal.rizwan45@yahoo.com (K.R.); sadiamalik6858@yahoo.com (S.N.); zubairmkn@yahoo.com (M.Z.); mahwishnadeem001@gmail.com (M.A.); 2 Department of Chemistry, University of Sahiwal, Sahiwal 57000, Pakistan; 3 Department of Chemistry, University of Education, Attock Campus, Attock 43600, Pakistan; i4hashmi@hotmail.com; 4 Faculty of Pharmacy, Universiti Teknologi MARA Cawangan Selangor Kampus Puncak Alam, Bandar Puncak Alam, Selangor 42300, Malaysia; 5 Atta-ur-Rahman Institute for Natural Products Discovery (AuRIns), Universiti Teknologi MARA Cawangan Selangor Kampus Puncak Alam, Bandar Puncak Alam, Selangor 42300, Malaysia

**Keywords:** suzuki coupling, cross-coupling, palladium, transition metal, imines, anilines, density functional theory

## Abstract

A series of (*E*)-4-bromo-*N*-((3-bromothiophen-2-yl)methylene)-2-methylaniline analogs synthesized in considerable yields through Suzuki cross-coupling reactions. Various electron donating and withdrawing functional moieties were successfully incorporated under the employed reaction conditions. Reaction of 4-bromo-2-methylaniline (**1**) with 3-bromothiophene-2-carbaldehyde (**2b**) in the existence of glacial acetic acid, provided (*E*)-4-bromo-*N*-((3-bromothiophen-2-yl)methylene)-2-methylaniline (**3b**) in excellent yield (94%). Suzuki coupling of **3b** with different boronic acids in the presence of Pd(PPh_3_)_4_/K_3_PO_4_ at 90 °C led to the synthesis of the monosubstituted and bisubstituted products **5a**–**5d** and **6a**–**6d** in moderate yields (33–40% and 31–46%, respectively). Density functional theory (DFT) investigations were performed on different synthesized analogues **5a**–**5d**, **6a**–**6d** to determine their structural characteristics. The calculations provide insight into the frontier molecular orbitals (FMOs) of the imine-based analogues and their molecular electrostatic potential (MESP). Reactivity descriptors like ionization energy (I), electron affinity (A), chemical hardness (*ƞ*) and index of nucleophilicity have been calculated for the first time for the synthesized molecules.

## 1. Introduction

Imines are flexible intermediates extensively used in the preparation of drugs, pesticides and other organic materials. These compounds, also known as Schiff bases or azomethines have a (-C=N-) functional group [1]. They are commonly used in the industrial, material and pharmacological domains. These compounds are known for their antifungal, antibacterial, anticancer, anticonvulsant, anti-inflammatory, analgesic, antimicrobial, anti-tubercular, anti-malarial, anti-HIV and antioxidant activities. Schiff bases based compounds have been employed as fungicide, pesticides, insecticide and bactericides [1,2,3,4,5,6,7]. They play a wide role in different fields of chemistry as bio-inorganic, catalysis, encapsulation, transport-separation and in magneto-chemistry [8], because their coordinative capability is excellent. Moreover, their capability to display photochromism (a color transformation after interacting with energy) enhances their applications in diverse fields such as adjusting and determining the intensity of radiation, imaging-systems and in optical computers [9]. Schiff bases also being used in various reaction systems as acid catalysts, oxidation and reduction catalysts. Schiff bases also show special attention for the metal ions [1].

Various research groups have reported different methodologies for the synthesis of Schiff bases [10]. They can be produced by the condensation of carbonyl compounds with amines. This method is widely used, but has a few associated drawbacks such as long reaction times and tedious work-ups [11,12]. Schmeyers and co-workers synthesized benzylidene aniline derivatives by grinding together solid anilines and benzaldehydes [13]. Varma and co-workers described the synthesis of enamines and imines by using clay as catalyst under microwave irradiation [14]. Various catalysts like clays, SiO_2_, P_2_O_5_, Al_2_O_3_, Ru(0), zeolites and other metrices have been employed previously for synthesis of Schiff bases with better yield [15,16,17,18].

In a previous report we described that when pyridine-based Schiff’s bases were synthesized and a Suzuki reaction was carried out the imine bond was hydrolysed. Computational studies revealed that this may be due to interaction of pyridine nitrogen with Pd (palladium) atoms of the Suzuki catalyst which led to hydrolysis of imine bond [19]. Then we carried another study using thiophene-containing Schiff bases in Suzuki coupling reactions and non-hydrolytic products were obtained [20]. To further examine imine bond hydrolysis in Suzuki reactions, in the present work, we again carried out the Suzuki coupling of thiophene-containing Schiff bases and this time we have incorporated methyl groups as well in Schiff bases. A novel series of thiophene-containing imines has been synthesized by Suzuki coupling reactions and their reactivity and structural characteristics have been explored by computational studies.

## 2. Results and Discussion

### 2.1. Chemistry

**Scheme 1:**
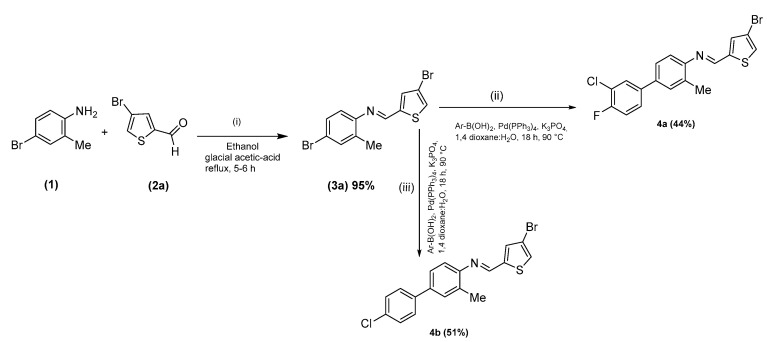
Synthesis of (*E*)-4-bromo-*N*-((4-bromothiophen-2-yl)methylene)-2-methylaniline (**3a**) and Suzuki coupling of **3a** with aryl boronic-acids. *Reagents and conditions*: (i) **1** (0.307 g, 1.612 mmol), **2a** (0.307 g, 1.612 mmol), ethanol (10 mL), glacial acetic-acid (5–6 drops) (ii) **3a** (0.200 g, 0.560 mmol), Pd(PPh_3_)_4_ (0.0321 g, 5-mol%), 3-chloro-4-fluorophenyl boronic acid (0.1165 g, 0.670 mmol), K_3_PO_4_ (0.280 g, 1.340 mmol) (iii) **3a** (0.330 g, 0.835 mmol), Pd(PPh_3_)_4_ (0.048 g, 5-mol%), 3-chlorophenyl boronic acid (0.160 g, 1.002 mmol), K_3_PO_4_ (0.420 g, 2.005 mmol).

In this study, Suzuki-coupling of 4-bromo-*N*-((4-bromothiophene-2-yl)methylene)aniline and 4-bromo-*N*-((3-phenylthiophene-2-yl)methylene)aniline with various arylboronic acids has been examined. In the first step, 4-bromo-2-methylaniline (**1**) reacted with the 4-bromothiophene-2-carbaldehyde (**2a**) in glacial acetic acid to obtain (*E*)-4-bromo-*N*-((4-bromothiophen-2-yl)methylene)-2-methylaniline (**3a**) in excellent yield (95%). Furthermore, in the second step the Suzuki reaction of **3a** was carried out using two different boronic-acids (3-chloro-4-fluorobenzene boronic acid and 4-chlorobenzene boronic acid) to afford the thiophene-based imines **4a**, **4b** in moderate yields of 44% and 51%, respectively (Scheme 1).

**Scheme 2:**
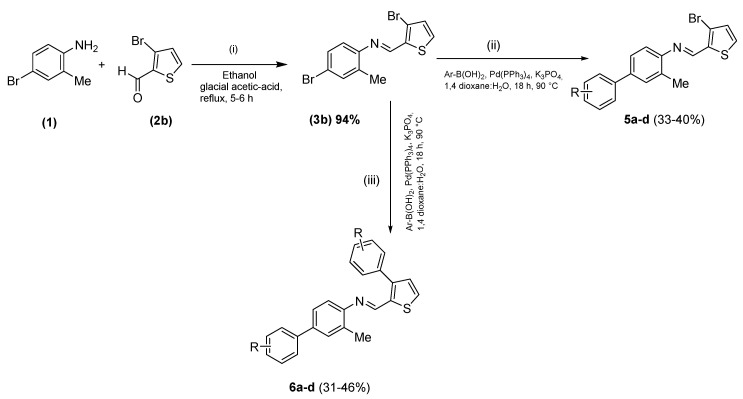
Synthesis of (*E*)-4-bromo-*N*-((3-bromothiophen-2-yl)methylene)-2-methylaniline (**3b**) and Suzuki-coupling of **3b** with aryl boronic-acids. *Reagents and conditions*: (i) **1** (0.307 g, 1.612 mmol), **2b** (0.307 g, 1.612 mmol), ethanol (10 mL), glacial acetic acid (5–6 drops) (ii) **3b** (0.150 g, 0.417 mmol), Pd(PPh_3_)_4_ (0.0241 g, 5-mol%), arylboronic acid (1 eq, 0.4178 mmol), K_3_PO_4_ (0.195 g, 0.918 mmol) (iii) **3b** (0.150 g, 0.417 mmol), Pd(PPh_3_)_4_ (0.0241 g, 5-mol%), arylboronic acid (2 eq, 0.4178 mmol), K_3_PO_4_ (0.177 g, 0.835 mmol).

In Scheme 2 the 2, 4-bromo-2-methylaniline (**1**) reacted with 3-bromothiophene-2-carbaldehyde (**2b**) in the existence of glacial acetic acid, consequently producing (*E*)-4-bromo-*N*-((3-bromothiophen-2-yl)methylene)-2-methylaniline (**3b**) in excellent yield (94%). In the next step, Suzuki-coupling of **3b** was carried out with different boronic acids (1 equivalent) to synthesize the monosubstituted products **5a**–**5d** in moderate yields (33–40%). The bromo group at the aryl moiety was preferentially regioselectively substituted because this is most active position as compared to the thiophenyl-Br position.

**Figure 1:**
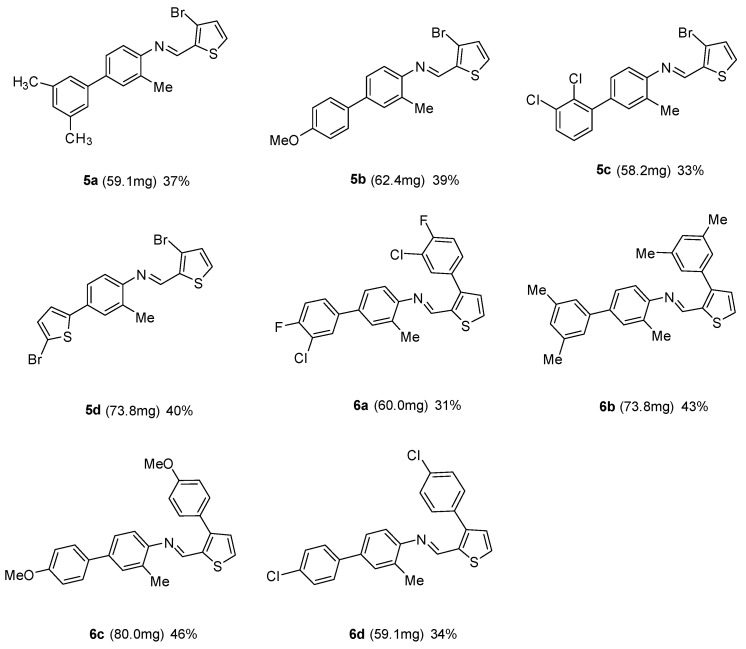
Substrate scope of Suzuki-coupling of (*E*)-4-bromo-*N*-((3-bromothiophen-2-yl)methylene)-2-methylaniline (**3b**) with various aryl and het-aryl boronic acids.

Lack of reactivity was observed with *ortho*-substituted arylboronic acids due to steric factors which hinder the in mechanism during the transmetalation step [21,22], and as a result low yields are obtained as in the cases of **5c** and **5d** (Figure 1). When **3b** was reacted with 2 equivalents of boronic acid, a double Suzuki coupling occurred and disubstituted products **6a**–**6d** were obtained in moderate yields (31–46%) (Figure 1). More bulky groups also lower the reaction rate and produce low yields as in the case of **6a** and **6d**. However, the appropriate selection of the catalyst (Pd source, ligand choice), solvent nature and amount of the base and temperature are fundamental for the success of these cross-coupling reactions. No imine bond hydrolysis was observed in both the monosubstituted and disubstituted products, which suggests that the thiophene sulphur atom does not interact with the Pd atom of the catalyst.

### 2.2. Density Functional Theory (DFT) Studies

**Figure 2:**
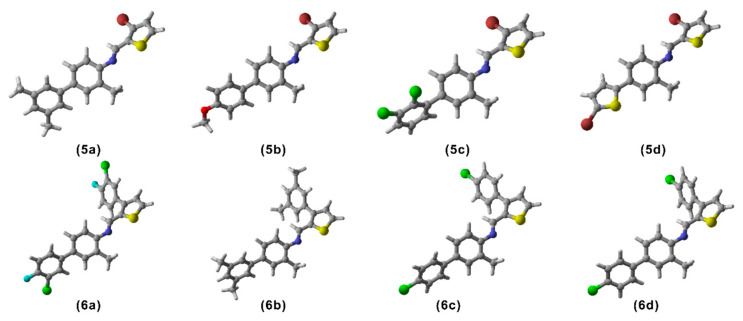
Optimized structures of Synthesized analogues (**5a**–**5d**, **6a**–**6d**) at PBE0-D3BJ/def2-TZVP/SMD_1,4-Dioxane_ level of theory. In 3D models, grey colour represents carbon (C), white represents hydrogens (H), green is for chlorine atoms (Cl), red colour represents oxygen (O), brown colour is for bromine (Br), light blue colour is for fluorine (F), and blue colour represents nitrogen (N) atoms.

All synthesized analogues **5a**–**5d** and **6a**–**6d** (Figure 2) were designed in GaussView and submitted for the optimization of geometry at the PBE0-D3BJ/def2-TZVP/SMD_1,4-Dioxane_ level of theory employing Gaussian 09.

A subsequent frequency calculation on each optimized geometry confirmed it as a true minimum by the absence of negative frequencies. The main objective of the DFT studies is to estimate the properties which are difficult to calculate experimentally and DFT calculations have proven to compute these properties like the frontier orbital analyses and other reactivity descriptors with a great accuracy.

#### 2.2.1. Frontier Molecular Orbital (FMO) Studies

**Figure 3:**
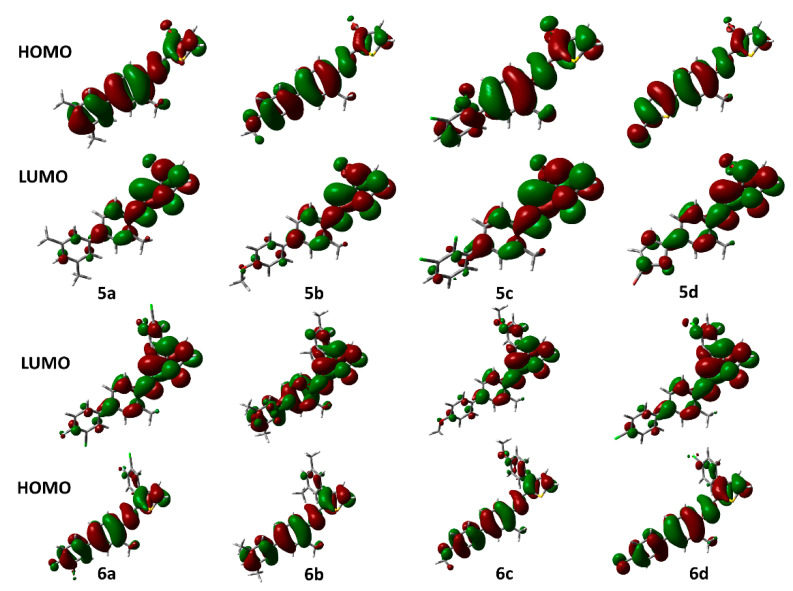
A presentation of the frontier orbitals of various synthesized analogues (**5a**–**5d, 6a**–**6d**) calculated at PBE0 D3BJ/def2-TZVP/SMD_1,4-Dioxane_ level of theory.

All chemical and physical characteristics of molecules depend mostly on the electrons in the molecule. Electronic structure calculations have become easy and accessible with the advent of modern desktop computers. DFT calculations provide us with a wealth of information about the electronic structure which includes the frontier molecular orbitals (FMOs). The electronic transitions between FMOs (HOMO and LUMO) can describe the chemical kinetics of a compound. FMO plots of the compounds under study have been given in Figure 3. The energies of HOMO, LUMO, and their gap (ΔE) have been presented in the Table 1. The energy gap (ΔE) values in Table 1 lie in a narrow range for all these molecules i.e., 3.72–4.15 eV. This means that the reactivity differences of **5a**–**5d**, **6a**–**6d** are not a lot. According to these values, compound **5c** should be the least reactive one (ΔE = 4.15 eV) while **5d** should be the most reactive one (ΔE = 3.72 eV). This can be explained on the basis of the manifestation of a 2,3-dichlorophenyl moiety on the central phenyl-ring as seen in Figure 2 (structure **5c**). Contrary to that, the presence of a thiophene ring instead of previously described dichlorophenyl, the electron density gets away from the methylated phenyl ring which makes it a more suitable electrophile.

The isodensity distribution in the FMOs of synthesized analogues **5a**–**5d**, **6a**–**6d** shows a similar behavior for all derivatives. The isodensity is primarily found on the aromatic rings and partially on the thiophene ring for the HOMO and it is heavily located on the thiophenes in the case of the LUMO with almost no isodensity on the methyl group. This isodensity uniformity makes the compounds stable and less reactive.

#### 2.2.2. Nonlinear Optical Properties

With nonlinear optics we can change the colour of a beam of light by passing it through a specific material and change its shape in space and time. Due to this possibility, materials capable of second-order nonlinear optical (NLO) properties have gathered attention of scientists. These materials are becoming common in different imaging techniques and sensors. DFT can successfully calculate the hyperpolarizability (*β*_tot_) values of the organic molecules which elaborate their capability to show electron push and pull mechanisms [23].

The synthesized compounds **5a**–**5d**, **6a**–**6d** have been studied for their hyperpolarizability values using DFT calculations (Table 1). Phenyl groups in these compounds generally act as electron acceptors with other groups as donors on them. Strong electron donors can push the electrons to the aromatic system causing them to flow through the system. In that scenario, compound **5d** has the highest *β* value with **5b** having the second highest *β* value. This can be described by the fact that a bromothiophene group shown in the left bottom in **5d** in Figure 2 is acting as a strong activating group while on its *para* position is the imine which is inductively electron withdrawing group and on the basis of these observations **5d** is predicted to be a good nonlinear optical material. Similarly, in **5b**, the methoxy group is an activating group and combined with an imine at the *para* position, it enhances the movement of electrons through the molecule, thus showing a good *β* value.

#### 2.2.3. Molecular Electrostatic Potential

**Figure 4:**
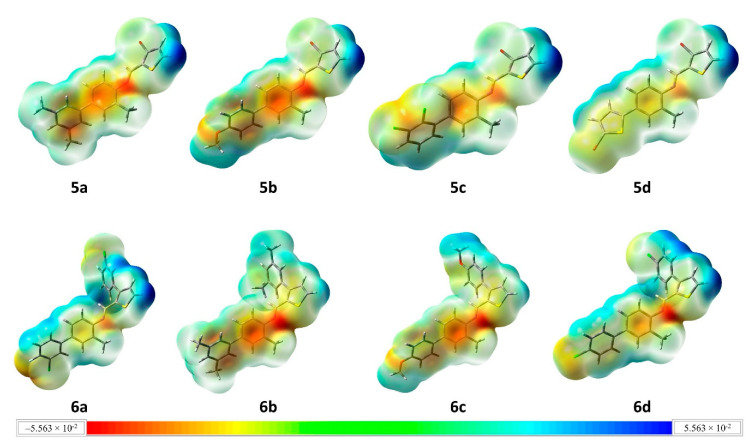
A presentation of all the molecular electrostatic potentials of all the molecules (**5a**–**5d**, **6a**–**6d**) calculated at PBE0-D3BJ/def2-TZVP/SMD_1,4-Dioxane_ level of theory. The scale below shows the colour coding. The values in the scale are in Hartrees.

Molecular electrostatic potential (MESP) graphs are very useful 3D plots of molecules that help us visualize the charge distribution, size, and shape of molecules. These describe the energy of a proton at its current position. By different colours, one can see the density of electrons at different positions of the molecule. The red colour shows the areas which are electron rich and are good nucleophilic sites while blue colour shows electron deficient regions that represent the electrophilic sites in the molecule. MESP plots of compounds **5a**–**5d** and **6a**–**6d** are given in Figure 4.

#### 2.2.4. Conceptual DFT Reactivity Descriptors

Conceptual DFT descriptors of reactivity may also be used in conjunction with the FMO energies to describe the reactivity of a molecule. The calculation of these electronic properties have been reported widely in the literature [23,24,25,26,27]. Ionization potential (*I*), electron affinity (*A*), chemical hardness (*ƞ*), and electronic chemical potential (*μ*) have been computed on the basis of Koopman’s theorem according to the formulas described previously by us [28]. The values of all these descriptors are presented in the Table 2.

As evident from the earlier FMO analysis, compound **5d** has been predicted to be the most reactive one. This result is clearly reproduced and supported by the chemical hardness (*ƞ*) value which is the lowest for **5d**. As *ƞ* value shows the ease of movement of electrons through the system, so its least value represents the greater activity of the compound (**5d**). Similar is the case for compound **5c** that has the highest *ƞ* value, thus making it the least reactive in the series. The other reactivity descriptor, electrochemical potential (*μ*) represents the tendency of a specie to distribute itself equally in the solution in a container so the higher value of *μ* will lead to lesser reactivity and vice versa.

## 3. Materials and Methods

### 3.1. General

Uncorrected melting points (m.p) were determined for the synthesized analogues. Proton and carbon NMR spectra were run in deuterochloroform (CDCl_3_) at 400/100 MHz on a spectrometer (400/100 MHz, Bruker, Billercia, MA, USA), respectively. To purify the compounds column chromatography utilizing silica gel of 70–230 mesh size was employed. Silica gel 60 PF _254_ plates (Merck, New York, NY, USA) were used to monitor the reactions and the product spots were visualized using 254 nm UV lamp. See Supplementary Material for spectra.

### 3.2. Generalized Methodology for the Synthesis of ***3a*** and ***3b***

A Schlenk flask was dried in the oven. Equimolar amounts of 4-bromo-2-methylaniline (**1**, 0.307 g, 1.612 mmol) and 4-bromothiophene carbaldehyde (**2a** or **2b**, 0.307 g, 1.612 mmol) were added and then the mixture refluxed for 8–10 h in existence of the ethanol (10 mL) and glacial acetic acid (CH_3_COOH, 5–6 drops). Reaction progression was followed with the help of TLC. After cooling the mixture, the precipitates formed were filtered. The products appeared as shiny yellow solids. For purification purposes, column chromatography was employed.

### 3.3. Generalized Methodology for the Suzuki Coupling of ***3a*** and ***3b***

In an oven-dried Schlenk flask that equipped with a magnetic stirbar, Schiff bases [(**3a,** 0.200 g, 0.560 mmol for synthesis of **4a**) and (**3a,** 0.330 g, 0.835 mmol for synthesis of **4b**)] or (**3b,** 0.150 g, 0.417 mmol for synthesis of **5a**–**5d** and **6a**–**6d**), solvent (1, 4-dioxane, 4 mL), and Pd(PPh_3_)_4_ catalyst (5 mol%) were added, then the mixture was stirred and refluxed (30 min) in the presence of an inert gas (argon). After 30 min K_3_PO_4_ and the appropriate aryl boronic acid (1 or 2 equivalents) and H_2_O (1 mL) were added up (for detailed amounts of all reactants see the captions of Scheme 1 and Scheme 2). Again the mixture was stirred for 18 h at 90 °C. Silica gel TLC plates were used to check the reaction progress. After completion the f obtained mixture was filtered and then further diluted by using ethyl acetate (CH_3_COOC_2_H_5_) solvent. For reaction work-up purposes distilled H_2_O was added. Separation of aqueous and organic layers was carried out with help of a separatory funnel, the organic layer was dried with magnesium sulphate and solvent evaporation carried out with rotary evaporator. Desired products were obtained as solids after column chromatography purification and further various characterization studies were carried for structure analysis.

### 3.4. Characterization Data

*(E)-4-Bromo-N-((4-bromothiophen-2-yl)methylene)-2-methylaniline* (**3a**): Yield = 546 mg (95%), m.p; 184–185 °C, ^1^H-NMR; (400 MHz, chloroform-d), δ: 7.42–7.38 (m, 3H-Aryl), 7.34–7.31 (m, 1H-thiophene), 7.28 (s, 1H-CH=N), 6.82 (d, *J* = 6.8 Hz, 1H-thiophene), 2.34 (s, 3H-Me), ^13^C-NMR; (100 MHz, chloroform-d), δ: 152.2, 146.2, 144.1, 135.2, 132.3, 129.5, 124.1, 123.0, 121.4. 121.0, 108.1, 17.1, EI-MS *m*/*z* (%):357.33 [M + H]; 358.23 [M + 2]; 360.33 [M + 4]; [M − Br] = 277.96, [M − 2Br] = 199.05, [M − CH_3_] = 184.02. Anal. Calcd for the; C_12_H_9_Br_2_NS: C, 40.14; H, 2.53, Found: C, 40.11; H, 2.51%. R_f_ = 0.65 (30% EtOAc in hexanes).

*(E)-4-Bromo-N-((3-bromothiophen-2-yl)methylene)-2-methylaniline* (**3b**): Yield = 540 mg (94%), m.p; 172–173 °C, ^1^H-NMR; (400 MHz, chloroform-d), δ: 7.40–7.28 (m, 3H-Aryl), 7.32–7.29 (m, 1H-thiophene), 7.28 (s, 1H CH=N), 6.83 (d, *J* = 6.8, 1H-thiophene), 2.36 (s, 3H-Me), ^13^C-NMR; (100 MHz, chloroform-d), δ: 152.1, 145.0, 144.2, 134.2, 131.3, 129.2, 124.2, 123.1, 121.0. 120.1, 108.0, 17.3. EI-MS *m*/*z* (%): 357.88 [M + H]; 358.23 [M + 2]; 360.33 [M + 4]; [M − Br] = 277.96, [M − 2Br] = 199.05, [M − CH_3_] = 184.02. Anal. Calcd for the; C_12_H_9_Br_2_NS: C, 40.14; H, 2.53, Found: C, 40.12; H, 2.56%. R_f_ = 0.67 (30% EtOAc in hexanes).

*(E)-N-((4-Bromothiophen-2-yl)methylene)-3′-chloro-4′-fluoro-3-methyl-[1,1′-biphenyl]-4-amine* (**4a**): Yield = 100.7 mg (44.2%), m.p; 78–79 °C, ^1^H-NMR; (400 MHz, chloroform-d), δ: 7.71–7.65 (m, 2H-Aryl,1H-CH=N), 7.50–7.37 (m, 3H-aryl), 7.20 (t, *J* = 7.0, 1H-aryl, 1H-thiophene), 7.04 (d, *J* = 7.0, 1H-thiophene), 2.31 (s, 3H-Me). ^13^C-NMR; (100 MHz, chloroform-d), δ: 156.1, 152.2, 145.4, 145.7, 140.2, 138.0, 132.0, 131.1, 129.9, 128.1, 126.2, 123.4, 122.1, 121.0, 119.2, 117.2, 107.4, 18.5. EI-MS *m*/*z* (%): 407.95 [M + H]; 408.95 [M + 2]; 410.55 [M + 4]; [M − Br] = 328.04, [M − Cl] = 293.04, [M − F] = 274.04, [M − CH_3_] = 259.05. Anal. Calcd for the; C_18_H_12_BrNSClF: C, 52.90; H, 2.96, Found: C, 52.93; H, 2.94%. R_f_ = 0.45 (25% EtOAc in hexanes).

*(E)-N-((4-Bromothiophen-2-yl)methylene)-4′-chloro-3-methyl-[1,1′-biphenyl]-4-amine* (**4b**): Yield = 166.4 mg (51.2%), m.p; 199–200 °C, ^1^H-NMR; (400 MHz, chloroform-d), δ: 7.71–7.65 (m, 2H-aryl, 1H-CH=N), 7.50–7.37 (m, 3H-Aryl), 7.22 (t, *J* = 7.0, 2H-Aryl, 1H-thiophene), 7.03 (d, *J* = 6.9, 1H-thiophene), 2.29 (s, 3H-Me). ^113^C-NMR; (100 MHz, chloroform-d), δ: 152.5, 146.4, 145.3, 140.4, 138.3, 133.1, 132.0, 131.0, 130.1, 129.1, 128.1, 126.1, 123.6, 122.1, 121.2, 108.5, 18.1. EI-MS *m*/*z* (%): 389.96 [M + H]; 390.96 [M + 2]; 392.96 [M + 4]; [M − Br] = 310.05, [M − Cl] = 275.08, [M − CH_3_] = 208.05. Anal. Calcd for the; C_18_H_13_BrNSCl: C, 55.33; H, 3.35, Found: C, 55.30; H, 3.32%. R_f_ = 0.49 (25% EtOAc in hexanes).

*(E)-N-((3-Bromothiophen-2-yl)methylene)-3,3′,5′-trimethyl-[1,1′-biphenyl]-4-amine* (**5a**): Yield = 59.1 mg (37%), m.p; 201–202 °C, ^1^H-NMR; (400 MHz, chloroform-d), δ: 8.75 (s, 1H-CH=N), 7.79–7.59 (m, 4H-Aryl), 7.51–7.33 (m, 2H-aryl, 2H-thiophene), 2.34 (s, 6H), 2.30 (s, 3H-Me). ^13^C-NMR; (100 MHz, chloroform-d), δ: 152.6, 145.4, 141.3, 140.0, 139.1, 138.0, 132.2, 131.3, 131.0, 129.3, 126.1, 125.9, 125.0, 124.2, 123.5, 122.9, 122.1, 21.1, 18.0. EI-MS *m*/*z* (%): 384.05[M + H]; 385.05[M + 2]; [M − Br] = 304.12, [M − CH_3_] = 289.09, [M − 2CH_3_] = 274.06, [M − 3CH_3_] = 259.05. Anal. Calcd for the; C_20_H_18_BrNS: C, 62.50; H, 4.72, Found: C, 62.48; H, 4.75%. R_f_ = 0.55 (35% EtOAc in hexanes).

*(E)-N-((3-Bromothiophen-2-yl)methylene)-4′-methoxy-3-methyl-[1,1′-biphenyl]-4-amine* (**5b**): Yield = 62.4 mg (39%), m.p; 183–184 °C, ^1^H-NMR; (400 MHz, chloroform-d), δ: 8.75 (s, 1H-CH=N), 7.78–7.60 (m, 4H-Aryl), 7.35–7.22 (m, 3H-aryl), 7.10–7.08 (m, 2H-thiophene), 3.78 (s, 3H), 2.34 (s, 3H). ^13^C-NMR; (100 MHz, chloroform-d), δ: 159.3, 152.1, 145.0, 140.0, 133.0, 132.0, 131.4, 126.1, 123.3, 122.6, 122.0, 121.2, 120.1, 119.2, 114.0, 110.1, 18.6, 55.0, 17.9. EI-MS *m*/*z* (%): 3845.04[M + H]; 386.05[M + 2]; [M − Br] = 306.8, [M − Br, CH_3_] = 291.091. Anal. Calcd for the; C_19_H_16_BrNOS: C, 59.07; H, 4.17, Found: C, 59.06; H, 4.15%. R_f_ = 0.52 (35% EtOAc in hexanes).

*(E)-N-((3-Bromothiophen-2-yl)methylene)-2′,3′-dichloro-3-methyl-[1,1′-biphenyl]-4-amine* (**5c**): Yield = 58.2 mg (33%), m.p; 168–169 °C, ^1^H-NMR; (400 MHz, chloroform-d), δ: 8.73 (s, 1H-CH=N), 7.75–7.59 (m, 4H-Aaryl), 7.35–7.20 (m, 2H-aryl, 2H-thiophene), 2.33 (s, 3H-Me). ^13^C-NMR; (100 MHz, chloroform-d), δ: 152.0, 145.8, 142.1, 134.1, 133.2, 132.0, 131.4, 131.1, 129.4, 127.5, 127.0, 126.1, 123.0, 122.9, 122.6, 122.1, 110.0, 18.6. EI-MS *m*/*z* (%): 423.91[M + H]; 424.90[M + 2]; 426.91[M + 4]; [M − Br] = 344, [M − Cl] = 309.04, [M − 2Cl] = 274.06, [M − CH_3_] = 259.04. Anal. Calcd for the; C_18_H_12_BrCl_2_NS: C, 50.85; H, 2.85, Found: C, 50.84; H, 2.81%. R_f_ = 0.48 (30% EtOAc in hexanes).

*(E)-4-(5-Bromothiophen-2-yl)-N-((3-bromothiophen-2-yl)methylene)-2-methylaniline* (**5d**): Yield = 73.8 mg (40.3%), m.p; 196–197 °C, ^1^H-NMR; (400 MHz, chloroform-d), δ: 8.67 (s, 1H-CH=N), 7.74–7.59 (m, 3H-aryl), 7.09 (d, *J* = 6.8 Hz, 2H-thiophene), 6.97 (d, *J* = 5.8 Hz, 2H-thiophene), 2.45 (s, 3H). ^13^C-NMR; (100 MHz, chloroform-d), δ: 152.0, 146.2, 140.1, 132.0, 131.9, 131.4, 131.1, 129.0, 125.0, 123.5, 122.9, 122.0, 121.0, 111.2, 110.0, 18.0. EI-MS *m*/*z* (%): 439.87[M + H]; 440.86[M + 2]; 442.81[M + 4]; [M − Br] = 359.91, [M − 2Br] = 281.03, [M − 2Cl] = 274.06, [M − CH_3_] = 266. Anal. Calcd for the; C_16_H_11_Br_2_NS_2_: C, 43.56; H, 2.51, Found: C, 43.52; H, 2.53%. R_f_ = 0.45 (35% EtOAc in hexanes).

*(E)-3′-Chloro-N-((3-(3-chloro-4-fluorophenyl)thiophen-2-yl)methylene)-4′-fluoro-3-methyl-[1,1′-biphenyl]-4-amine* (**6a**): Yield = 60.0 mg (31.4%), m.p; 181–182 °C, ^1^H-NMR; (400 MHz, chloroform-d), δ: 8.72 (s, 1H-CH=N), 7.53–7.51 (m, 3H-aryl), 7.49–7.47 (m, 3H-aryl), 7.30–7.27 (m, 3H-aryl), 6.78 (d, *J* = 7.8 Hz, 2H-thiophene), 2.38 (s, 3H-Me), ^13^C-NMR; (100 MHz, chloroform-d), δ: 158.6, 157.2, 152.5, 145.0, 140.1, 137.0, 136.1, 133.0, 132.0, 131.6, 130.1, 129.9, 129.3, 128.1 128.9, 126.2, 122.7, 121.3, 121.2, 117.4, 18.6. EI/MS *m*/*z* (%): 458.01[M + H]; 459[M + 2]; 461.01[M + 4]; [M − F] = 438.01, [M − 2F] = 384.04, [M − Cl] = 403.06, [M − 2Cl] = 349.09, [M − CH_3_] = 334.05. Anal. Calcd for the; C_24_H_15_Cl_2_F_2_NS: C, 62.89; H, 3.30, Found: C, 62.86; H, 3.33%. R_f_ = 0.43 (35% EtOAc in hexanes).

*(E)-N-((3-Bromothiophen-2-yl)methylene)-3-methyl-[1,1′-biphenyl]-4-amine* (**6b**): Yield = 73.8 mg (43.3%), m.p; 183–185 °C, ^1^H-NMR; (400 MHz, chloroform-d), δ: 8.90 (s, 1H-CH=N), 7.98 (d, *J* = 8.9, 2H-Aryl), 7.62–7.60 (m, 4H-Aryl), 7.25–7.20 (m, 3H-Aryl), 6.73 (d, *J* = 6.7, 2H-thiophene), 2.39 (s, 12H-4Me), 2.33 (s, 3H-Me), ^13^C-NMR; (100 MHz, chloroform-d), δ: 152.2, 145.2, 141.0, 140.0, 138.6, 138.0, 136.2, 136.1, 132.0, 131.0, 130.8, 130.2, 129.8, 126.2, 125.5, 121.3, 120.6, 21.8, 18.0. EI-MS *m*/*z* (%): 410.17[M + H]; 411.18[M + 2]; 413.18[M + 4]; [M − CH_3_] = 394.16, [M − 2CH_3_] = 379.13, [M − 3CH_3_] = 364.11, [M − 4CH_3_] = 349.09, [M − 5CH_3_] = 334.05. Anal. Calcd for the; C_28_H_27_NS: C, 82.11; H, 6.64, Found: C, 82.09; H, 6.60%. R_f_ = 0.55 (35% EtOAc in hexanes).

*(E)-N-((3-(3,5-Dimethylphenyl)thiophen-2-yl)methylene)-3,3′,5′-trimethyl-[1,1′-biphenyl]-4-amine* (**6c**): Yield = 80 mg (46.4%), m.p; 207–209 °C, ^1^H-NMR; (400 MHz, chloroform-d), δ: 8.70 (s, 1H-CH=N), 7.95 (d, *J* = 7.5 Hz, 2H-Aryl), 7.53–7.50 (m, 7H-Aryl), 7.36 (d, *J* = 6.9 Hz, 2H-aryl), 6.73 (d, *J* = 6.7 Hz, 2H-thiophene), 3.78 (s, 6H-OMe), 2.54 (s, 3H-Me). ^13^C-NMR; (100 MHz, chloroform-d), δ: 160.2, 159.5, 152.2, 140.1, 145.3, 136.4, 133.1, 132.0, 131.0, 130.1, 129.8, 129.0, 128.7, 126.2, 122.7, 121.3, 120.6, 114.8, 55.0, 18.1. EI-MS *m*/*z* (%): 414.12[M + H]; 415[M + 2]; 417.13[M + 4]; [M − CH_3_] = 398.15, [M − OCH_3_] = 367.10, [M − 2OCH_3_] = 336.05. Anal. Calcd for the; C_26_H_23_NO_2_S: C, 75.52; H, 5.61, Found: C, 75.50; H, 5.64%. R_f_ = 0.63 (30% EtOAc in hexanes).

*(E)-4′-Chloro-N-((3-(4-chlorophenyl)thiophen-2-yl)methylene)-3-methyl-[1,1′-biphenyl]-4-amine* (**6d**): Yield = 59.1 mg (33.7%), m.p; 164–166 °C, ^1^H-NMR; (400 MHz, chloroform-d), δ: 8.70 (s, 1H-CH=N)), 7.86 (d, *J* = 8.5 Hz, 3H-Aryl), 7.70–7.68 (m, 5H-Aryl), 7.43–7.40 (m, 3H-aryl), 6.53 (d, *J* = 5.7 Hz, 2H-thiophene), 2.56 (s, 3H-Me), ^13^C-NMR; (100 MHz, chloroform-d), δ: 152.3, 145.0, 140.0, 138.5, 136.2, 134.0, 134.1, 133.2, 132.3, 131.1, 130.1, 129.0, 128.0, 126.1, 122.2, 121.1, 120.2, 18.1. EI-MS *m*/*z* (%): 422.06[M + H]; 423.05[M + 2]; 425.03[M + 4]; [M − CH_3_] = 406.02, [M − Cl] = 371.05, [M − 2Cl] = 336.07. Anal. Calcd for the; C_24_H_17_Cl_2_S: C, 68.25; H, 4.06, Found: C, 68.21; H, 4.07%. R_f_ = 0.68 (35% EtOAc in hexanes).

### 3.5. Computational Methods

Computations have been performed using density functional theory (DFT) on the Gaussian 09 (Revision D.01) software [29]. Optimizations led to the minimum energy structures which were then confirmed to be true minima on the potential energy surface by subsequent frequency calculations and imaginary-frequency was absent. Adamo’s hybrid version [30] of Perdew, Burke, and Ernzerhof-functional (PBE0) [31] was used in all the calculations aided by Grimme’s empirical dispersion correction (D3) with Becke-Johnston damping (D3BJ) [32,33,34]. Basis set employed was triple ζ basis set, def2-TZVP [35]. The implicit solvation effects were modeled using Polarizable Continuum Model (PCM) with the integral equation formalism variant (IEFPCM) [36,37,38,39,40,41,42] with Cramer and Truhlar’s [43] SMD parameter set as implemented in Gaussian 09 [29]. The solvent used in calculations was 1,4-dioxane in all the calculations. For visualization and 3D image generation the GaussView 5.0.9 and CYLview [44] tools have been used.

## 4. Conclusions

A series of (*E*)-4-bromo-*N*-((3-bromothiophen-2-yl)methylene)-2-methylaniline analogs **5a**–**5d** and **6a**–**6d** have been synthesized in moderate yields (33–40% and 31–46%, respectively) via Suzuki cross-coupling reactiona. Various functional electron donating and withdrawing moieties were accepted under the reaction conditions. DFT investigations of the synthesized compounds have been done to study their structural and electronic properties. After a detailed insight into FMOs of these compounds and their different reactivity descriptors, it is concluded that compound **5d** is the most reactive one while **5c** is the most stable in the series. NLO calculations demonstrate **5d** and **5b** as having good potential to act as potential nonlinear optical materials.

## Figures and Tables

**Table 1:** HOMO, LUMO Energies and band gap. All the HOMO and LUMO energies are given in eV. Hyperpolarizability (*β*) values are given in Hartrees.

Compound	E*_HOMO_*	E*_LUMO_*	HOMO-LUMO Gap	Hyperpolarizability
**5a**	−5.95	−2.01	3.94	4218.78
**5b**	−5.75	−2.00	3.76	9932.64
**5c**	−6.23	−2.08	4.15	1460.58
**5d**	−5.88	−2.16	3.72	7588.64
**6a**	−6.09	−2.09	4.00	2709.57
**6b**	−5.81	−1.84	3.97	2751.87
**6c**	−5.98	−2.03	3.95	3232.40
**6d**	−5.96	−2.02	3.94	3605.95

**Table 2:** Reactivity descriptors (*I*, *A*, *η*, *µ*, *ω*) of the synthesized analogues compounds under study.

Compounds	Ionization Potential, *I* (eV)	Electron Affinity, *A* (eV)	Chemical Hardness, *ƞ* (eV)	Electronic Chemical Potential, *μ* (eV)	Electrophilicity Index, *ω* (eV)
**5a**	5.95	2.01	−1.97	3.98	−4.03
**5b**	5.75	2.00	−1.88	3.87	−4.00
**5c**	6.23	2.08	−2.07	4.16	−4.16
**5d**	5.88	2.16	−1.86	4.02	−4.35
**6a**	6.09	2.09	−2.00	4.09	−4.18
**6b**	5.81	1.84	−1.99	3.82	−3.68
**6c**	5.98	2.03	−1.98	4.01	−4.06
**6d**	5.96	2.02	−1.97	3.99	−4.04

## Supplementary Materials

The Supplementary Materials are available online.
