# Supplementary material for: Palladium(0) Catalyzed Synthesis of (E)-4-Bromo-N-((3-bromothiophen-2-yl)methylene)-2-methylaniline Derivatives via Suzuki Cross-Coupling Reaction: An Exploration of Their Non-Linear Optical Properties, Reactivity and Structural Features
Source: Molecules. 2021 Sep 15;26(18):5605. doi: 10.3390/molecules26185605 (PMC8465532; doi:10.3390/molecules26185605)
Supplement: Supplementary file 1 [file molecules-26-05605-s001.zip › molecules-1012835-supplementary.pdf]

HNMR S#1 R1, CDCL3

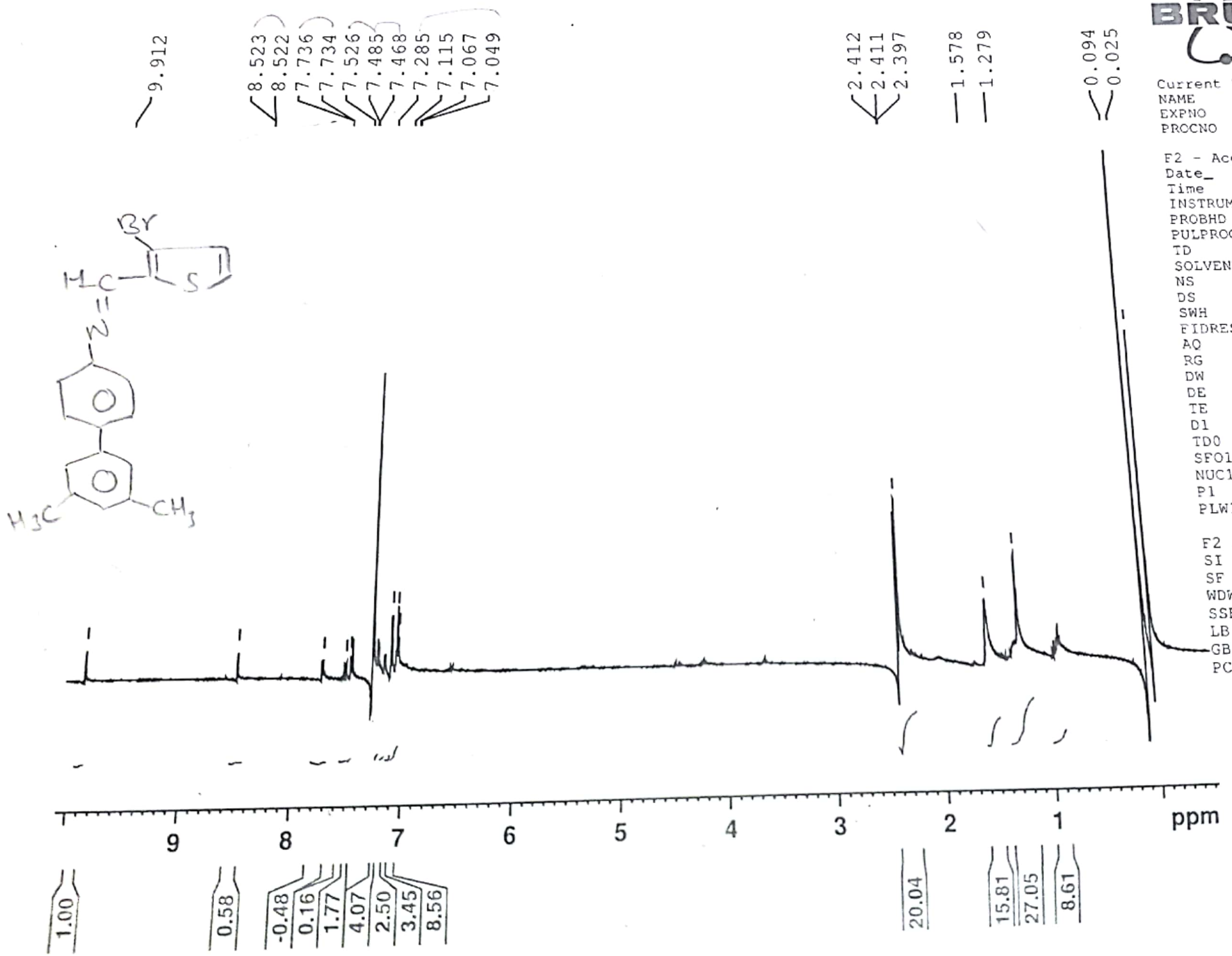

BRUKER

Current Data Parameters

NAME	20160726-5
EXPNO	1
PROCNO	1

F2 - Acquisition Parameters

Date_	20160726
Time	14.27 h
INSTRUM	spect
PROBHD	Z119470_0240 (
PULPROG	zg30
TD	65536
SOLVENT	CDCl3
NS	16
DS	2
SWH	10000.000 Hz
FIDRES	0.305176 Hz
AQ	3.2767999 sec
RG	150.69
DW	50.000 usec
DE	6.50 usec
TE	298.0 K
D1	1.00000000 sec
TD0	1
SFO1	500.2930893 MHz
NUC1	1H
P1	10.00 usec
PLW1	20.81399918 W

F2 - Processing parameters

SI	65536
SF	500.2900000 MHz
WDW	EM
SSB	0
LB	0.30 Hz
GB	0
PC	1.00

Scanned with CamScanner

CNMR S#1 R1, CDCL3

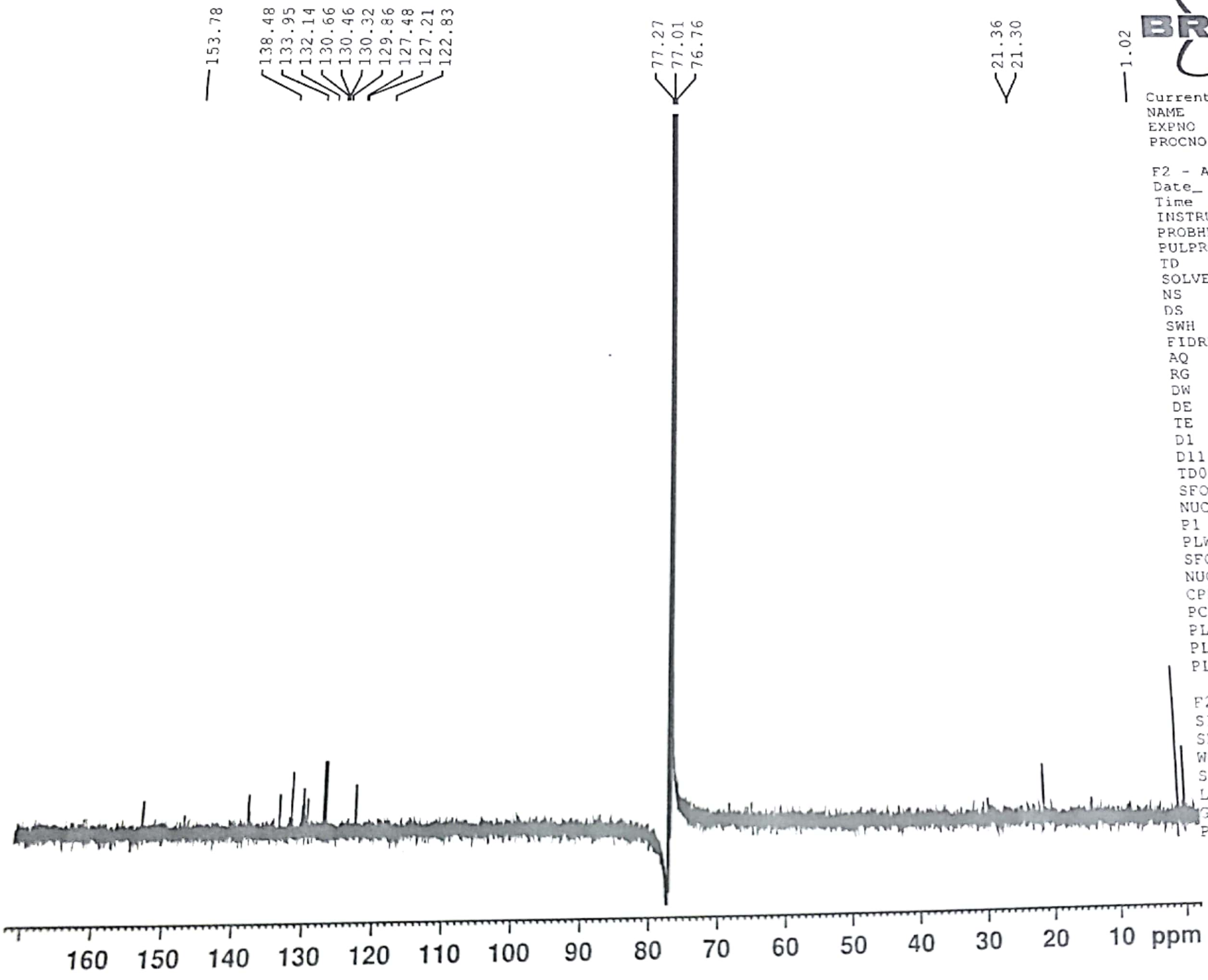

Current Data Parameters

NAME	20160726-6
EXPNO	2
PROCNO	1

F2 - Acquisition Parameters

Date_	20160726
Time	15.25 h
INSTRUM	spect
PROBHD	Z119470_0240 (
PULPROG	zgpg30
TD	65536
SOLVENT	CDCl3
NS	1024
DS	4
SWH	29761.904 Hz
FIDRES	0.908261 Hz
AQ	1.1010048 sec
RG	189
DW	16.800 usec
DE	6.50 usec
TE	298.0 K
D1	2.00000000 sec
D11	0.03000000 sec
TD0	1
SFO1	125.8106004 MHz
NUC1	13C
P1	10.00 usec
PLW1	88.97299957 W
SFO2	500.2920012 MHz
NUC2	1H
CPDPRG[2	waltz16
PCPD2	80.00 usec
PLW2	20.81399918 W
PLW12	0.31764621 W
PLW13	0.15920430 W

F2 - Processing parameters

SI	32768
SF	125.7980206 MHz
WDW	EM
SSB	0
LB	1.00 Hz
GB	0
PC	1.40

Scanned with CamScanner

CNMR S#1 R1, CDCL3

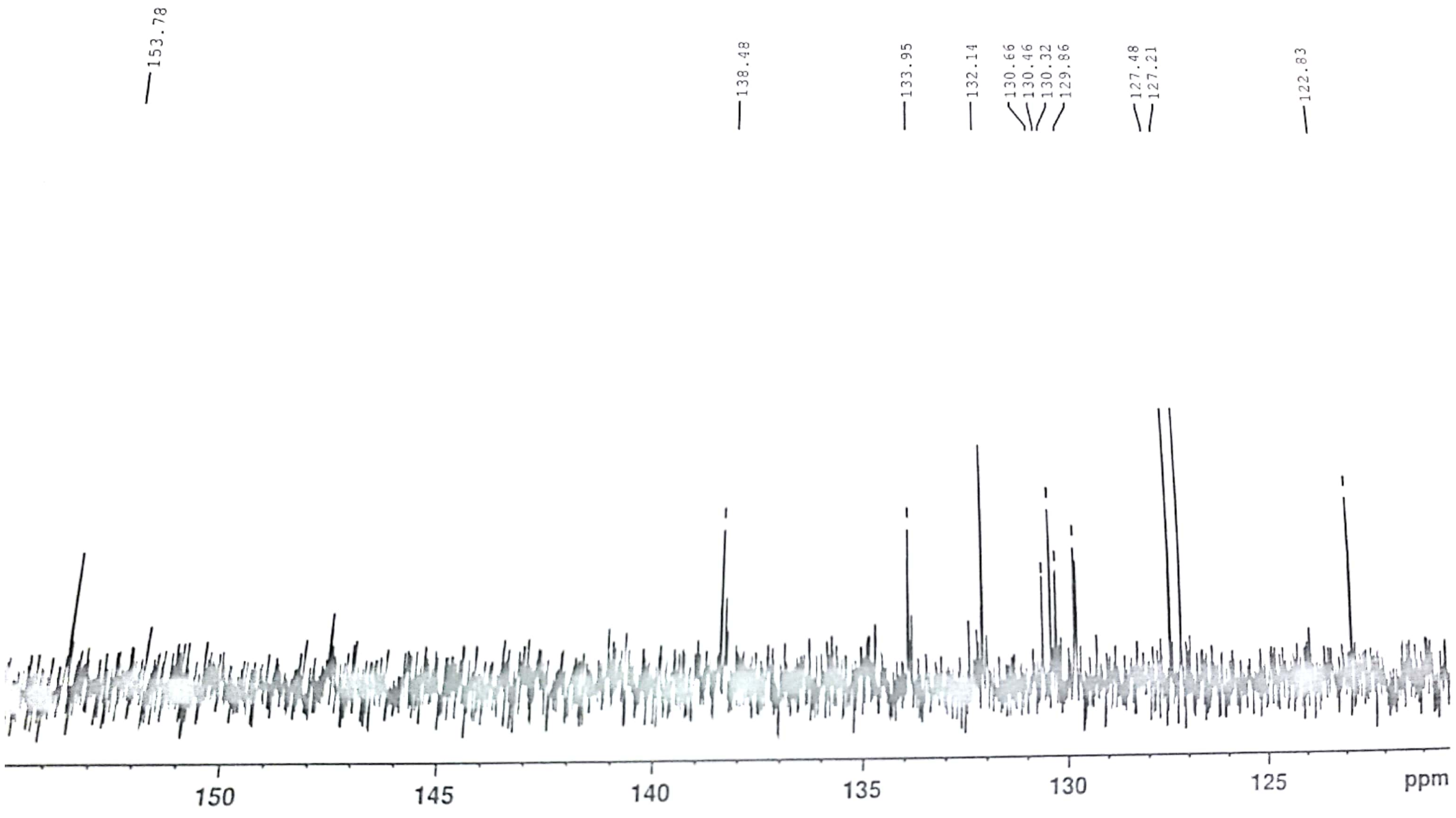

Scanned with CamScanner
